# The Emergence of NMDA Receptor Metabotropic Function: Insights from Imaging

**DOI:** 10.3389/fnsyn.2016.00020

**Published:** 2016-07-28

**Authors:** Kim Dore, Jonathan Aow, Roberto Malinow

**Affiliations:** ^1^Center for Neural Circuits and Behavior, Department of Neuroscience and Section for Neurobiology, Division of Biology, University of California at San DiegoSan Diego, CA, USA; ^2^Genome Institute of SingaporeSingapore, Singapore

**Keywords:** ion-flux independent, FRET-FLIM, long-term depression (LTD), NMDAR interactions with CaMKII, amyloid-beta induced depression, PP1, excitotoxicity

## Abstract

The NMDA receptor (R) participates in many important physiological and pathological processes. For example, its activation is required for both long-term potentiation (LTP) and long-term depression (LTD) of synaptic transmission, cellular models of learning and memory. Furthermore, it may play a role in the actions of amyloid-beta on synapses as well as in the signaling leading to cell death following stroke. Until recently, these processes were thought to be mediated by ion-flux through the receptor. Using a combination of imaging and electrophysiological approaches, ion-flux independent functions of the NMDAR were recently examined. In this review, we will discuss the role of metabotropic NMDAR function in LTD and synaptic dysfunction.

## Introduction

Transmembrane receptors have traditionally been divided into two classes: ionotropic and metabotropic. Ionotropic glutamate receptors (iGluRs) form channels that allow the passage of ions into the cell to drive signaling, while metabotropic glutamate receptors (mGluRs) generate downstream effects without ion-flux. The boundary between these two classes is not completely distinct, as there has been evidence that several iGluRs are capable of producing effects in the absence of ion-flux. For example, the N-terminal domain of GluA2, a subunit of the AMPA receptor (AMPAR), is sufficient to promote spine formation in hippocampal neurons (Passafaro et al., 2003). Another iGluR, the kainate receptor, can modulate GABA transmission without ion-flux (Rodríguez-Moreno and Lerma, 1998).

The NMDA receptor (NMDAR), a member of the iGluR family, is ubiquitously expressed and plays numerous roles in the brain (Traynelis et al., 2010). Given its ability to conduct calcium ions (Ca^2+^) well, it has been assumed that downstream signaling triggered by NMDARs was mediated by Ca^2+^ influx and increased cytoplasmic Ca^2+^. However, to allow Ca^2+^ entry through the receptor, several conditions have to be fulfilled: (1) glutamate must bind to GluN2 subunits; (2) glycine, the co-agonist must bind to GluN1 subunits; and (3) neurons must be sufficiently depolarized to eliminate the voltage-dependent magnesium ion (Mg^2+^) block of the channel. During high-frequency stimulation (HFS) these three conditions are met resulting in long-term potentiation (LTP; Bliss and Collingridge, 1993). For long-term depression (LTD), however, the role of the NMDAR is not as clear. A long-standing model has proposed that while LTP requires a large increase in cytoplasmic Ca^2+^, a moderate rise in cytoplasmic Ca^2+^ would produce LTD (Lisman, 1989; Malenka, 1994). However, several recent studies indicate that NMDARs can induce LTD without ion-flow through the receptor (Carter and Jahr, 2016; Nabavi et al., 2013; Dore et al., 2015; Stein et al., 2015). Other publications have shown that excitotoxicity as well as amyloid-beta-induced synaptic depression depend on NMDAR activity but are likewise independent of ion-flow (Kessels et al., 2013; Tamburri et al., 2013; Birnbaum et al., 2015; Weilinger et al., 2016). In this review, we will discuss how these studies probed NMDAR metabotropic activity with an emphasis on the imaging techniques used.

## NMDAR-Dependent LTD can be Induced Independently of Ion-Flux

**Figure 1:**
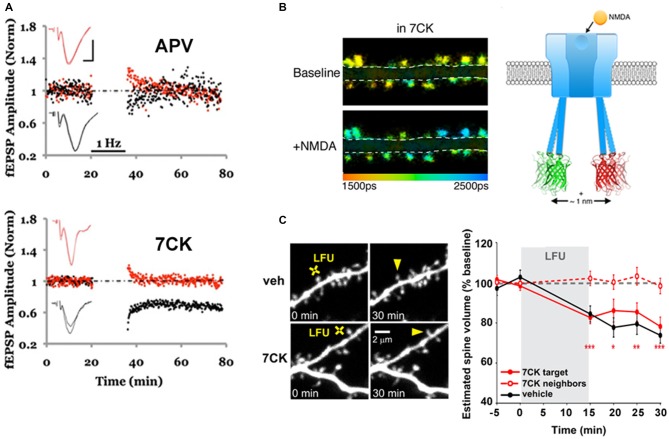
**Ion-flux independent long-term depression (LTD). (A)** APV, but not 7-chloro-kynurenate (7CK), blocks LTD. LTD (1 Hz, black bar) was delivered to the test pathway (black symbols), while the control pathway (red symbols) received no stimulus; the slice was incubated in APV (top) or 7CK (bottom) throughout the experiment. Note that there was no change to the control or test pathway in APV, as expected, whereas the test pathway was significantly depressed after LTD induction in 7CK; representative of *N* = 15, APV, *p* > 0.05 LTD; *N* = 15, 7CK, *p* < 0.01 LTD. Insets: superimposed traces obtained before and after LTD in the test (black line) and control (red line) pathways. Scale bars 0.3 mV, 5 ms. Modified from Nabavi et al. (2013). **(B)** Chemical LTD induction (+NMDA) resulted in spines that exhibit less Förster Resonance Energy Transfer (FRET; and higher GFP fluorescence lifetimes; ps = picoseconds) between the GFP- and mCherry-tagged GluN1 subunits (see Toolbox), indicating movement of the NMDAR cytoplasmic domain. Modified from Dore et al. (2015). **(C)** A low frequency glutamate uncaging protocol (LFU), similar to the 1 Hz electrical stimulation in **(A)**, induced spine shrinkage both in vehicle or 7CK-incubated GFP-expressing neurons, indicating that ion-flux through the NMDAR is not required for spine shrinkage. Modified from Stein et al. (2015).

Interestingly, evidence for ion-flux independent LTD can be observed in data from older literature. Over 20 years ago, data were published indicating that MK-801, which blocks NMDAR channels, blocked LTP but failed to block LTD (Mayford et al., 1995). A similar effect was obtained by a different group (Scanziani et al., 1996). Surprisingly, these observations were not discussed in either study. The ion-flux dependence of LTD was recently examined more closely (Nabavi et al., 2013). Low-frequency stimulation (LFS) produced LTD in the presence of either MK-801 or 7-chloro-kynurenate (7CK, a competitive GluN1 antagonist; see Figure 1A) but not APV (a competitive GluN2 antagonist); all three antagonists effectively blocked ion-flux through the NMDAR. Moreover, LTD was observed in experiments in which intracellular Ca^2+^ was clamped to basal levels, suggesting that a rise in intracellular Ca^2+^ is not required for LTD. It was thus proposed that glutamate binding to the NMDAR could induce a conformational change in the cytoplasmic domain of the NMDAR that triggers downstream signaling resulting in LTD.

To test if ligand binding could drive movement of the NMDAR intracellular domain, FRET-FLIM [Forster resonance energy transfer measured by fluorescence lifetime imaging of the FRET donor, see Toolbox and (Wallrabe and Periasamy, 2005; Yasuda, 2006)] was employed (Dore et al., 2015). Recombinant GluN1 subunits of the NMDAR were tagged with GFP or mCherry at their carboxyl(c)-terminus and co-expressed in neurons. As the magnitude of FRET is very sensitive to the distance and orientation of the interacting fluorophores, nanometer-scale changes in distance can be reliably detected. Bath application or uncaging of glutamate in the presence of MK-801 or 7CK, but not APV, produced a transient change in FRET consistent with conformational movement of the NMDAR cytoplasmic domain (Figure 1B). Infusing neurons with a GluN1 c-terminus antibody through a patch pipette blocked the ligand-driven FRET change as well as LTD, suggesting that this conformational change is required for LTD induction.

Toolbox **FRET measurements using fluorescence lifetime imaging microscopy.**

**Figure:**
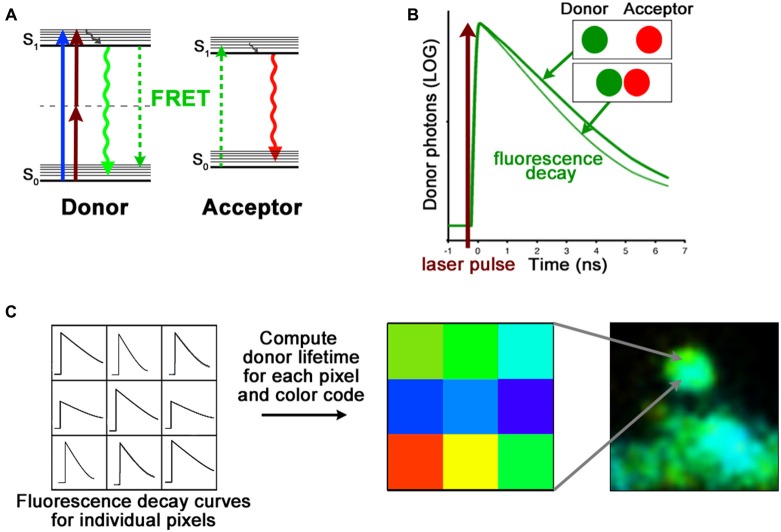
FRET is a non-radiative energy transfer mechanism between two fluorescent molecules. There are two main requirements for successful FRET. First the fluorescence emission of the FRET donor must overlap with the FRET acceptor absorption spectrum; and second, these fluorescent molecules must be no more than ~10 nm apart from each other (Lakowicz, 2006). This spatial requirement of FRET is very sensitive; it can thus be used as a “molecular ruler” to assess protein structure (Gustiananda et al., 2004) or to monitor subtle conformational changes (Dore et al., 2015). FRET can be measured by acquiring a series of images in different combinations of excitation and emission channels or by photobleaching of the FRET acceptor. However, these methods are prone to errors and are generally not well suited for measurements in living cells expressing fluorescent proteins (Selvin, 2000; Yasuda, 2006; Piston and Kremers, 2007). Fluorescence lifetime is defined as the average time a molecule stays in its excited state before emitting a photon (A). Because lifetime is an intrinsic property of fluorophores, it is independent of experimental conditions such as concentration, excitation intensity and photobleaching (Lakowicz, 2006; Yasuda, 2006). Importantly, by adding an additional route for the donor fluorophore to return to ground state, the degree of FRET makes the fluorescence lifetime of the donor proportionally shorter (A,B). To measure fluorescence lifetimes, the most common approach is time correlated single photon counting (TCSPC; Becker et al., 2004) which calculates the time delay between the detection of fluorescence photons and laser excitation pulses (B). When TCSPC is combined with laser scanning microscopy, it is possible to obtain fluorescence lifetimes, and hence detect changes in donor-acceptor distances, at every pixel of an image (B,C). **FRET-FLIM. (A)** Jablonski diagram of FRET donor and acceptor energy levels. After excitation by a 1-photon (blue arrow) or 2-photon (brown arrows) laser, the FRET donor can return to ground state by emitting a photon (green arrow) or by transferring its energy to a nearby acceptor (dashed green arrows). **(B)** The fluorescence lifetime of the FRET donor, which becomes shorter with increased proximity of the FRET acceptor, is calculated with the fluorescence decay curve. **(C)** Time-correlated single photon counting (TCSPC) records fluorescence decay curves for each pixel of an image. By fitting these curves, the fluorescence lifetime of the FRET donor can be assessed. The FLIM image is then color coded according to the FRET donor lifetime at each pixel.

Downstream signaling events were also examined using FRET-FLIM (Aow et al., 2015). Protein-phosphatase 1 (PP1) is one of the first molecules whose activity was shown to be required for LTD (Mulkey et al., 1993) and it co-immunoprecipitates with the NMDAR complex (Husi et al., 2000). FRET between GluN1-GFP and PP1-mCherry, observed in baseline conditions, was transiently reduced during chemical LTD induction. This ligand-driven decrease in FRET required NMDAR conformational movement but not PP1 activity (Aow et al., 2015). It is possible that the transient movement of PP1 relative to the NMDAR cytoplasmic domain exposes the catalytic active site of PP1 to a target unavailable under basal conditions. One potential target is calcium-calmodulin dependent protein kinase II (CaMKII; Strack et al., 1997), which is recruited to the NMDAR complex during LTP stimuli (Otmakhov et al., 2004) and whose activity is required for both LTP (Malenka et al., 1989; Malinow et al., 1989; Silva et al., 1992) and LTD (Coultrap et al., 2014). By monitoring FRET between fluorescently-tagged GluN1 and CaMKII, a delayed decrease in the NMDAR-CaMKII interaction was observed during ion-flux independent LTD (Aow et al., 2015). This effect depended on PP1 activity and was not seen with a CaMKII mutant that cannot be dephosphorylated at Thr-286 (CaMKII-Thr-286-Asp), suggesting that dephosphorylation of Thr-286 is necessary to modify the NMDAR-CaMKII interaction (Aow et al., 2015). Co-immunoprecipitation experiments additionally revealed that the amount of total CaMKII bound to the NMDAR was unaffected by ion-flow independent LTD, whereas levels of phosphorylated Thr-286 were reduced both during and after LTD induction (Aow et al., 2015). These results are consistent with a model for ion-flux independent LTD in which glutamate binding to the NMDAR induces a conformational change in the NMDAR intracellular domain that facilitates PP1 access to and dephosphorylation of CaMKII at Thr-286, thereby repositioning the CaMKII holoenzyme within the NMDAR complex. The relocated CaMKII could in turn potentially act on a novel site of the GluA1 subunit (Ser-567) that undergoes phosphorylation during LTD (Coultrap et al., 2014). Consistent with this model, CaMKII phosphorylation of GluA1-Ser-567 does not require Ca^2+^ or calmodulin (Coultrap et al., 2014). Ultimately, this process could increase AMPAR endocytosis (Lüscher et al., 1999; Lin et al., 2000; Kim et al., 2001; Shi et al., 2001) and lead to depressed synaptic transmission.

Ion-flow independent NMDAR activation of downstream signaling pathways has also been linked to shrinkage of dendritic spines (Stein et al., 2015). Stein et al. used 2-photon laser scanning microscopy (TPLSM) to monitor structural changes in the dendritic spines of GFP-expressing hippocampal neurons. Low-frequency uncaging of glutamate produced a ~20% decrease in spine size that was independent of ion-flow through the NMDAR (Figure 1C). While high-frequency glutamate uncaging produced an increase in spine volume, the same stimulus in the presence of either 7CK or MK-801 led to spine shrinkage. This result is consistent with the finding that HFS (delivered electrically) in the presence of MK-801 produces LTD instead of LTP (Nabavi et al., 2013). Spine shrinkage was also abolished when p38 MAPK activity was blocked (Stein et al., 2015), which is again consistent with the observation that levels of phosphorylated p38 (which is the active form) were increased during ion-flow independent LTD (Nabavi et al., 2013). In the future, it will be important to elucidate how the initial movement in the NMDAR cytoplasmic domain subsequently affects signaling molecules, such as cofilin, calcineurin and p38, implicated in LTD.

An ion-flux-independent mechanism for NMDAR-dependent LTD has been challenged by some recent studies (Babiec et al., 2014; Volianskis et al., 2015; Sanderson et al., 2016) but confirmed by others (Kim et al., 2015; Stein et al., 2015; Carter and Jahr, 2016). It is notable that the experimental conditions used in these recent studies that failed to detect ion-flux-independent LTD were not identical to those supporting this form of LTD. For instance, NMDAR antagonists were typically acutely washed in and then out of the preparation during LTD induction (Volianskis et al., 2015), instead of being present throughout the experimental duration. Furthermore, control pathways, which monitor transmission onto the same neurons but do not receive the conditioning stimulus, were generally not included (Babiec et al., 2014; Volianskis et al., 2015; Sanderson et al., 2016). These differences in methodology are significant and can make an impact in the outcome and interpretation of results (Nabavi et al., 2014). Therefore, it will be important to compare carefully the experimental conditions employed by different studies. Nevertheless, it remains possible that two different, independent forms of NMDAR-dependent LTD exist: one that requires ion-flow through NMDARs and one that does not. Different experimental conditions could selectively recruit either of these two forms.

## Trafficking of NMDAR is Regulated by Synaptic Activity but not Ion-Flux

In addition to its more recently described role in LTD, a few older studies have indicated that ligand binding to the NMDAR, in the absence of ion-flux, could control NMDAR trafficking (Vissel et al., 2001; Barria and Malinow, 2002; Nong et al., 2003). The Westbrook lab showed that even with its pore blocked, ligand binding to the NMDAR drove tyrosine dephosphorylation of GluN2A subunits, resulting in NMDAR endocytosis and decreased NMDA currents. Another group separately observed that an initial application of glycine was sufficient to prime NMDARs for subsequent use-dependent endocytosis, again leading to a decline in NMDA currents (Nong et al., 2003). Moreover, synaptic replacement of GluN2B- with GluN2A-containing NMDARs, an important developmentally controlled process (Hestrin, 1992; Monyer et al., 1994; Sheng et al., 1994; Stocca and Vicini, 1998; Tovar and Westbrook, 1999), required ligand binding without ion flux (Barria and Malinow, 2002). Interestingly, the replacement of synaptic GluN2B-containing NMDARs by newly synthetized GluN2B-containing NMDARs did not require ligand binding. It is important to note that these effects on NMDAR trafficking required both agonist and co-agonist binding to NMDARs as they were blocked by antagonists to the glutamate binding site on GluN2 subunits or to the glycine binding site on GluN1 subunits (Vissel et al., 2001; Barria and Malinow, 2002; Nong et al., 2003); in contrast, LTD only requires ligand binding to GluN2 subunits (Nabavi et al., 2013).

## Metabotropic NMDAR Activity can Induce Synaptic Dysfunction

Recent results have suggested a role for metabotropic NMDAR activity in amyloid-beta mediated synaptic dysfunction, which may contribute to hippocampal deficits in Alzheimer’s disease (AD) and precede neurological symptoms by a decade or more (Terry et al., 1991; Reiman et al., 1996). A number of studies using electrophysiology and imaging have reported that amyloid-beta impairs LTP, depresses synaptic transmission and induces synapse loss in various hippocampal preparations (Chapman et al., 1999; Larson et al., 1999; Walsh et al., 2002; Wang et al., 2002; Kamenetz et al., 2003; Snyder et al., 2005; Hsieh et al., 2006; Shankar et al., 2007; Wei et al., 2010; Birnbaum et al., 2015). The effect of intracellularly delivered amyloid-beta is not clear, as one report indicated synaptic depression (Ripoli et al., 2014) while another indicated synaptic potentiation (Whitcomb et al., 2015). In many of these studies the electrophysiological results could be corroborated using imaging. For instance, Wei et al. (2010) used TPLSM to show that GFP-filled dendritic spines close to axons or dendrites overexpressing amyloid-beta displayed a smaller increase in spine volume following a chemically-induced LTP protocol as compared to more distant spines, suggesting that secreted amyloid-beta impaired LTP. Hsieh et al. (2006) used TPLSM of AMPARs tagged with the pH-sensitive GFP-variant SEP (Super-Ecliptic-Phluorin) to measure surface AMPARs and found that amyloid-beta reduced surface GluA1 and GluA2. Likewise, immunostaining and imaging primary cultures treated with amyloid-beta revealed a reduction in surface NMDARs (Snyder et al., 2005) and AMPARs (Almeida et al., 2005; Alfonso et al., 2014). The decrease in synaptic AMPAR and NMDAR currents correlates, therefore, with a decrease in surface receptors as determined with optical techniques. Finally, several groups have shown using TPLSM or confocal microscopy that endogenously expressed or exogenously applied amyloid-beta reduces spine density in GFP-expressing neurons (Hsieh et al., 2006; Shrestha et al., 2006; Calabrese et al., 2007; Shankar et al., 2007; Wei et al., 2010; Zempel et al., 2010), which may explain the electrophysiologically observed decreased frequency of miniature excitatory postsynaptic currents (Kamenetz et al., 2003; Hsieh et al., 2006). Therefore, amyloid-beta induces synaptic insults that can be directly observed through imaging.

**Figure 2:**
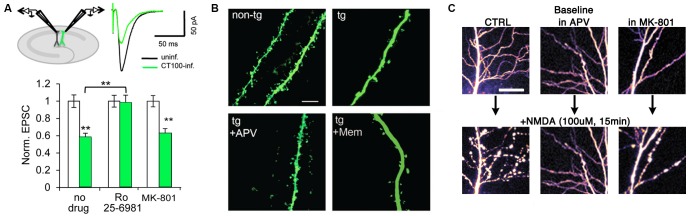
**Metabotropic NMDAR activity can induce synaptic dysfunction. (A)** APP-CT100 overexpression depresses synaptic transmission without ion-flux. Model figure for a dual whole-cell recording of an infected and a neighboring uninfected CA1 neuron (top left) and an example trace of evoked AMPA receptor (AMPAR) currents of such a recording (top right). Bottom, results from paired-recordings performed in ACSF containing no drug (*N* = 41), Ro 25-6981 (*N* = 37) or MK-801 (*N* = 25); **indicates *p* < 0.001. Modified from Kessels et al. (2013). **(B)** Spine density is reduced in a transgenic mouse model of Alzheimer’s disease (AD), APV blocks the effect but not memantine. Scale bar, 5 μm. Modified from Birnbaum et al. (2015). **(C)** Sustained NMDA application causes excito-toxic insults to CA1 neurons in the form of blebbing of dendrites (left panels). This effect is blocked by APV and CGP-78608 (middle panels) but not MK-801 (right panels). Scale bar, 25 μm. Modified from Weilinger et al. (2016).

A mechanism proposed to account for synaptic impairment by amyloid-beta is enhanced ionotropic glutamate receptor endocytosis. Indeed, there is evidence that inhibiting endocytic signaling pathways or overexpressing mutant endocytic-resistant receptors can ameliorate the reduction in AMPAR and/or NMDAR currents (Snyder et al., 2005; Hsieh et al., 2006; Knafo et al., 2016). Notably, Kessels et al. (2013) reported that despite block of ion flux, not all NMDAR antagonists prevented amyloid-beta-induced depression of AMPAR-mediated transmission. The GluN2 antagonists (R)-CPP, Ro25-6981, and ifenprodil afforded a complete block; whereas the GluN1 antagonist 7CK and the NMDAR pore blocker MK-801 had no effect (Figure 2A; Kessels et al., 2013). Thus, the block of depression correlated with actions on different NMDAR subunits rather than block of ion-flux. In another model, amyloid-beta oligomers exogenously applied to organotypic hippocampal slices acutely depressed AMPAR-mediated transmission in a manner that was dependent on synaptic stimulation and NMDAR activation but not NMDAR ion-flux (Tamburri et al., 2013). Both studies therefore suggest that amyloid-beta activates a metabotropic NMDAR signaling pathway that depresses synaptic transmission. The evidence that this pathway could then be involved in eventual spine loss comes from three other studies using imaging techniques. Two studies (Shankar et al., 2007, 2008) showed using TPLSM that (R)-CPP prevented amyloid-beta-induced spine loss in GFP-expressing organotypic slice neurons. Birnbaum et al. (2015) subsequently demonstrated that the competitive GluN2 antagonist APV also blocked spine loss in transgenic AD mice (as well as in hippocampal slices incubated in amyloid-beta oligomers), whilst MK-801, memantine, another NMDAR pore blocker, and buffering postsynaptic calcium ions with BAPTA had no effect (Figure 2B). That study (Birnbaum et al., 2015) also showed an amyloid-beta-induced reduction in PSD-95 and synaptophysin levels that was blocked by APV but not by MK-801 or memantine. Moreover, they demonstrated that p38 MAPK phosphorylation was increased by amyloid-beta in a NMDAR ion-flux independent manner and that spine loss depended on p38 MAPK activity (Birnbaum et al., 2015), supporting a link, previously examined (Hsieh et al., 2006), between LTD and amyloid-beta-induced depression. Taken together, these imaging results are in agreement with electrophysiological experiments and support the hypothesis that amyloid-beta toxicity depresses synaptic transmission via metabotropic NMDAR signaling that results in eventual spine loss.

The role of NMDARs in mediating excitotoxicity has been extensively described (reviewed in Choi, 1992), and it has been widely suggested that excessive Ca^2+^ influx through the receptor is responsible for inducing cell death (Choi, 1995; Tu et al., 2010). Interestingly, recent findings suggest that a metabotropic NMDAR “signalsome”—involving the NMDAR, the pannexin-1 channel (Panx1) and src kinase—is capable of inducing cellular dysfunction in response to excessive NMDAR stimulation (Weilinger et al., 2016). TPLSM was used to image fluorescently labeled CA1 neurons in acute rat hippocampal slices treated with a high dose of NMDA. This protocol induced blebbing in the dendrites of CA1 neurons as well as mitochondrial dysfunction, an effect that was blocked by co-application of the GluN1 antagonist CGP-78608 and APV, but not by MK-801, indicating that ligand binding to the NMDAR was capable of damaging neuronal morphology independently of ion flux through the receptor (Figure 2C). Interestingly, Panx1 channels appear to be located almost exclusively at the PSD (Zoidl et al., 2007), which suggests that this metabotropic NMDAR “signalsome” is synaptic. Additional experiments demonstrated that ligand binding, but not NMDAR ion flux, was necessary for downstream activation of Panx1 (Thompson et al., 2006, 2008; Weilinger et al., 2012). Metabotropic NMDAR activity did not change the degree of interaction between GluN1 and Panx1, but it did increase Src kinase binding to GluN1, Src activation, as well as Src-dependent phosphorylation of Panx1. Peptides that either disrupted the GluN1-Src interaction (Src_48_) or interfered with Panx1 phosphorylation (Tat-Panx_308_) were neuroprotective *in vitro*, and injection of Tat-Panx_308_ reduced brain lesion volume in an *in vivo* model of stroke. Indeed, as ischemia in the brain is believed to drive subsequent excitotoxicity, these results suggest that targeting Src or Panx1 in a clinical setting could be therapeutically effective.

## Concluding Remarks

**Figure 3:**
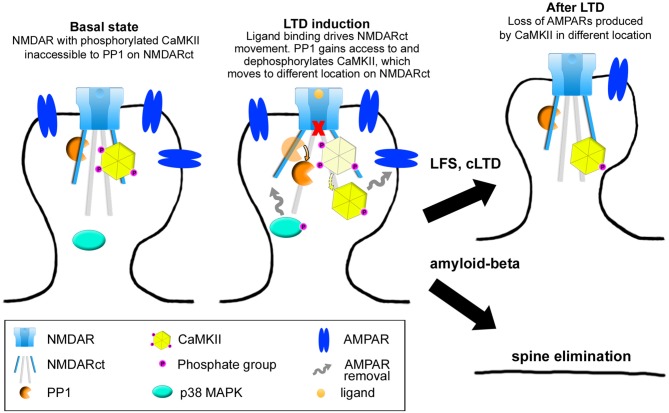
**Model of NMDAR metabotropic functions.** In baseline conditions, the NMDAR interacts with both protein-phosphatase 1 (PP1) and calcium-calmodulin dependent protein kinase II (CaMKII) which is phosphorylated at Thr286. Upon ligand binding, movement in the NMDAR cytoplasmic domain permits PP1 catalytic access to phospho-CaMKII-T286, this movement also inducing activation and phosphorylation of p38 MAPK. These signaling molecules (along with others) will eventually lead to AMPAR removal. In the context of LTD, these events will produce spine shrinkage whereas in the case of sustained amyloid-beta presence, spine elimination will occur.

A number of studies have provided evidence that physiological and pathological processes can be triggered by ligand binding to the NMDAR, without requiring flow of ions through its pore. It will be important to determine conditions that control whether an ionotropic or metabotropic NMDAR mechanism is engaged during LTD. Ion-flux independent LTD appears to be mediated by a movement in the NMDAR cytoplasmic domain that affects its interactions with at least two signaling proteins, PP1 and CaMKII. Subsequent signaling, with increased p38 MAPK phosphorylation likely, leads to AMPAR removal, shrinkage of dendritic spines and depressed synaptic transmission (Figure 3). Interestingly, it seems that if the stimulus recruiting metabotropic NMDAR function is sustained, as in the contexts of amyloid-beta overproduction or excitotoxic conditions following ischemia, metabotropic NMDAR activity can also lead to synaptic and neuronal dysfunction.

## Author Contributions

KD, JA and RM wrote the manuscript. KD designed figures.

## Conflict of Interest Statement

The authors declare that the research was conducted in the absence of any commercial or financial relationships that could be construed as a potential conflict of interest.
